# Draft genome sequencing of a multidrug-resistant *Klebsiella pneumoniae* strain MBBL2 isolated from mastitic cow milk

**DOI:** 10.1128/mra.01204-24

**Published:** 2025-01-29

**Authors:** Md. Morshedur Rahman, Naim Siddique, Kh. Yeashir Arafat, Ziban Chandra Das, Tofazzal Islam, M. Nazmul Hoque

**Affiliations:** 1 Molecular Biology and Bioinformatics Laboratory, Department of Gynecology, Obstetrics and Reproductive Health, Bangabandhu Sheikh Mujibur Rahman Agricultural University (BSMRAU) 198780 , Gazipur, Bangladesh; 2 Institute of Biotechnology and Genetic Engineering, BSMRAU 198780 , Gazipur, Bangladesh; The University of Arizona, Tucson, Arizona, USA

**Keywords:** draft genome, *Klebsiella pneumoniae*, multidrug resistance, virulence factors, mastitis, dairy cows

## Abstract

Milk from cows with mastitis is a primary source of bacteria harboring antibiotic resistance genes (ARGs), including *Klebsiella pneumoniae*. We present the genome sequence of *K. pneumoniae* strain MBBL2 isolated from mastitic cow milk, which contains numerous ARGs and virulence-associated genes potentially pathogenic to humans.

## ANNOUNCEMENT

*Klebsiella pneumoniae* is an opportunistic, gram-negative, encapsulated, non-motile pathogenic bacterium that can cause infections in both animals and humans (1, 2). It is commonly found in food, soil, water, plants, and on the skin and in the intestine of animals and humans (3, 4). It is a frequent cause of clinical mastitis (CM) in dairy cows and exhibits a high propensity for developing antimicrobial resistance (5, 6). These factors raise significant public health concerns and underscore the necessity for more extensive studies and the implementation of effective mastitis control and antibiotic stewardship strategies to mitigate its spread and impact.

Milk samples (10 mL) were collected from cows diagnosed with CM using the California Mastitis Test (7) at dairy farms in the Gazipur District (24.09°N, 90.41°E) of Bangladesh. A 100 µL aliquot of each collected milk sample was immediately added to 10 mL of nutrient broth (Biolife, Italy) and incubated overnight at 37°C (6). The enriched broth (2 µL) was then spread onto MacConkey Agar (HiMedia, India) at 37°C for 24 h. The phenotypic identification of *K. pneumoniae* was conducted based on colony morphology (pink color, circular) and Gram staining (gram-negative), while species level identification was performed using VITEK-2 System v9.01 (8). DNA extraction was carried out using the QIAamp DNA Mini Kit (QIAGEN, Germany) following the published protocol (9, 10). In brief, a single colony of the *K. pneumoniae* MBBL2 isolate from nutrient agar plate was inoculated into nutrient broth, incubated overnight at 37°C, and harvested by centrifugation prior to DNA extraction. The Nextera DNA Flex Kit (Illumina, USA) was utilized to construct libraries from 1 ng of high-purity DNA, followed by whole-genome sequencing on an Illumina MiSeq sequencer with a 2 × 250 bp protocol (11). The raw reads (*N* = 3,199,784; 464.3 Mbp; 100× coverage) were trimmed to remove Illumina adapters, known Illumina artifacts, and phiX reads using Trimmomatic v.0.39 (12), and quality was checked in FastQC v0.11.7 (13). The genome assembly and annotation were conducted using SPAdes v.3.15.5 (14) and National Center for Biotechnology Information Prokaryotic Genome Annotation Pipeline v.6.6 (15), respectively. Furthermore, CheckM v1.2.3 (16) was utilized for assessing the completeness of the *K. pneumoniae* MBBL2 genome. In the MBBL2 genome, ARGs were predicted by CARD (17), plasmids by PlasmidFinder (18), and VFGs by VFDB (19) executed in ABRicate (https://github.com/tseemann/abricate) on Linux command line program. Additionally, subsystem features were identified by RAST v.2.0 (20) and likely to be human pathogens by PathogenFinder v.1.1 (21). Default parameters were used for all software, unless otherwise specified.

The *K. pneumoniae* strain MBBL2 genome characteristics are given in Table 1. The draft genome of MBBL2 was about 5.4 Mbp (5,391,382 bp), with 57.5% guanine–cytosine contents and 100× coverage. Importantly, *K. pneumoniae* MBBL2 was found to possess 40 ARGs, six plasmids, 61 VFGs, and 393 subsystems related to different metabolic pathways/genes. Notably, the high pathogenicity score of 0.799 with a similarity to 97 pathogenic families indicates that this strain is potentially pathogenic not only to dairy cows but also to humans and other animals, highlighting its significant implications for dairy industry and public health.

**TABLE 1:** Genomic features of the *K. pneumoniae* strain MBBL2 isolated from raw milk of a cow diagnosed with clinical mastitis

*Klebsiella pneumoniae* strain MBBL2	
GenBank accession	JBINJS000000000
BioSample accession	SAMN44249570
SRA accession	SRR30951066
Assembled genome size (bp)	5,391,382
Guanine–cytosine %	57.5
Coverage (×)	100
Genome completeness (%)	98.55 (0.82% contamination)
N_50_ value (bp)	263,293
Number of contigs	84
Genes (total)	5,341
CDSs (total)	5,252
Genes (coding)	5,131
CDSs (with protein)	5,131
Genes (RNA)	89
rRNAs	1, 1, 1 (5S, 16S, 23S)
Complete rRNAs	1, 1 (5S, 16S)
tRNAs	74
ncRNAs	12
Pseudo genes (total)	121
CDSs (without protein)	121
Pseudo genes (ambiguous residues)	0 of 121
Pseudo genes (frameshifted)	59 of 121
Pseudo genes (incomplete)	67 of 121
Pseudo genes (internal stop)	24 of 121
Pseudo genes (multiple problems)	25 of 121
CRISPR arrays	1
Number of ARGs	40
Number of VFGs	61
Number of subsystems	393
Pathogenicity score	0.79
Matched with pathogenic families	97

## Data Availability

The whole genome shotgun project of the *K. pneumoniae* strain MBBL2 has been deposited in GenBank and the NCBI Sequence Read Archive (SRA) under BioProject accession number PRJNA1171635 and SRA accession number SRR30951066. The version described in this paper is version .1.

## References

[1] Cheng F, Li Z, Lan S, Liu W, Li X, Zhou Z, Song Z, Wu J, Zhang M, Shan W. 2018. Characterization of Klebsiella pneumoniae associated with cattle infections in southwest China using multi-locus sequence typing (MLST), antibiotic resistance and virulence-associated gene profile analysis. Braz J Microbiol 49 Suppl 1:93–100. doi:10.1016/j.bjm.2018.06.004 30150085 PMC6328855

[2] Hoque MN, Faisal GM, Moyna Z, Islam MS, Das ZC, Islam T. 2023. Draft genome sequence of a multidrug-resistant Klebsiella pneumoniae fecal isolate from a cow with clinical mastitis. Microbiol Resour Announc 12:e0073023. doi:10.1128/MRA.00730-23 37902381 PMC10652979

[3] Wareth G, Neubauer H. 2021. The animal-foods-environment interface of Klebsiella pneumoniae in Germany: an observational study on pathogenicity, resistance development and the current situation. Vet Res 52:16. doi:10.1186/s13567-020-00875-w 33557913 PMC7871605

[4] Wu M, Li X. 2015. *Klebsiella pneumoniae* and *Pseudomonas aeruginosa*, p 1547–1564. In Molecular medical microbiology. Elsevier.

[5] Munoz MA, Ahlström C, Rauch BJ, Zadoks RN. 2006. Fecal shedding of Klebsiella pneumoniae by dairy cows. J Dairy Sci 89:3425–3430. doi:10.3168/jds.S0022-0302(06)72379-7 16899675

[6] Hoque MN, Istiaq A, Clement RA, Gibson KM, Saha O, Islam OK, Abir RA, Sultana M, Siddiki AZ, Crandall KA, Hossain MA. 2020. Insights Into the Resistome of Bovine Clinical Mastitis Microbiome, a Key Factor in Disease Complication. Front Microbiol 11:860. doi:10.3389/fmicb.2020.00860 32582039 PMC7283587

[7] Hoque MN, Das ZC, Talukder AK, Alam MS, Rahman A. 2015. Different screening tests and milk somatic cell count for the prevalence of subclinical bovine mastitis in Bangladesh. Trop Anim Health Prod 47:79–86. doi:10.1007/s11250-014-0688-0 25326717

[8] Kumaran D, Laflamme C, Ramirez-Arcos S. 2023. A multiphasic approach to solve misidentification of Cutibacterium acnes as Atopobium vaginae during routine bacterial screening of platelet concentrates using the VITEK 2 system. Access Microbiol 5:000539. doi:10.1099/acmi.0.000539.v3 PMC10323807 37424557

[9] Rahman MM, Siddique N, Akter S, Das ZC, Hoque MN. 2024. Draft genome sequencing of Pediococcus pentosaceus strains isolated from cow milk. Microbiol Resour Announc 13:e0023824. doi:10.1128/mra.00238-24 38619270 PMC11080533

[10] Rahman M, Siddique N, Rahman AA, Das ZC, Islam T, Hoque MN. 2024. Whole-genome sequencing of Enterococcus faecalis probiotic strains isolated from raw milk of healthy cows. Microbiol Resour Announc 13:e0046524. doi:10.1128/mra.00465-24 39162453 PMC11385455

[11] Sultana T, Siddique N, Rahaman M, Rahman MM, Rahman AA, Talukder AK, Das ZC, Hoque MN. 2024. Draft genome sequencing of multidrug-resistant Pseudomonas asiatica strains isolated from dairy cows with clinical mastitis and their farm environment. Microbiol Resour Announc 13:e0090724. doi:10.1128/mra.00907-24 39404440 PMC11556121

[12] Bolger AM, Lohse M, Usadel B. 2014. Trimmomatic: a flexible trimmer for Illumina sequence data. Bioinformatics 30:2114–2120. doi:10.1093/bioinformatics/btu170 24695404 PMC4103590

[13] Andrews S. 2010. FastQC: a quality control tool for high throughput sequence data. Babraham Bioinformatics, Babraham Institute, Cambridge, United Kingdom.

[14] Bankevich A, Nurk S, Antipov D, Gurevich AA, Dvorkin M, Kulikov AS, Lesin VM, Nikolenko SI, Pham S, Prjibelski AD, Pyshkin AV, Sirotkin AV, Vyahhi N, Tesler G, Alekseyev MA, Pevzner PA. 2012. SPAdes: a new genome assembly algorithm and its applications to single-cell sequencing. J Comput Biol 19:455–477. doi:10.1089/cmb.2012.0021 22506599 PMC3342519

[15] Tatusova T, DiCuccio M, Badretdin A, Chetvernin V, Nawrocki EP, Zaslavsky L, Lomsadze A, Pruitt KD, Borodovsky M, Ostell J. 2016. NCBI prokaryotic genome annotation pipeline. Nucleic Acids Res 44:6614–6624. doi:10.1093/nar/gkw569 27342282 PMC5001611

[16] Parks DH, Imelfort M, Skennerton CT, Hugenholtz P, Tyson GW. 2015. CheckM: assessing the quality of microbial genomes recovered from isolates, single cells, and metagenomes. Genome Res 25:1043–1055. doi:10.1101/gr.186072.114 25977477 PMC4484387

[17] Jia B, Raphenya AR, Alcock B, Waglechner N, Guo P, Tsang KK, Lago BA, Dave BM, Pereira S, Sharma AN, Doshi S, Courtot M, Lo R, Williams LE, Frye JG, Elsayegh T, Sardar D, Westman EL, Pawlowski AC, Johnson TA, Brinkman FSL, Wright GD, McArthur AG. 2017. CARD 2017: expansion and model-centric curation of the comprehensive antibiotic resistance database. Nucleic Acids Res 45:D566–D573. doi:10.1093/nar/gkw1004 27789705 PMC5210516

[18] Carattoli A, Zankari E, García-Fernández A, Voldby Larsen M, Lund O, Villa L, Møller Aarestrup F, Hasman H. 2014. In Silico detection and typing of plasmids using plasmidfinder and plasmid multilocus sequence typing . Antimicrob Agents Chemother 58:3895–3903. doi:10.1128/AAC.02412-14 24777092 PMC4068535

[19] Chen L, Zheng D, Liu B, Yang J, Jin Q. 2016. VFDB 2016: hierarchical and refined dataset for big data analysis-10 years on. Nucleic Acids Res 44:D694–7. doi:10.1093/nar/gkv1239 26578559 PMC4702877

[20] Overbeek R, Olson R, Pusch GD, Olsen GJ, Davis JJ, Disz T, Edwards RA, Gerdes S, Parrello B, Shukla M, Vonstein V, Wattam AR, Xia F, Stevens R. 2014. The SEED and the rapid annotation of microbial genomes using subsystems technology (RAST). Nucleic Acids Res 42:D206–14. doi:10.1093/nar/gkt1226 24293654 PMC3965101

[21] Cosentino S, Voldby Larsen M, Møller Aarestrup F, Lund O. 2013. PathogenFinder-distinguishing friend from foe using bacterial whole genome sequence data. PLoS ONE 8:e77302. doi:10.1371/journal.pone.0077302 24204795 PMC3810466

